# STAT3 Silencing and TLR7/8 Pathway Activation Repolarize and Suppress Myeloid-Derived Suppressor Cells From Breast Cancer Patients

**DOI:** 10.3389/fimmu.2020.613215

**Published:** 2021-02-19

**Authors:** Elham Safarzadeh, Ali Mohammadi, Behzad Mansoori, Pascal H. G. Duijf, Shahryar Hashemzadeh, Vahid Khaze, Tohid Kazemi, Afshin Derakhshani, Nicola Silvestris, Behzad Baradaran

**Affiliations:** ^1^ Department of Microbiology and Immunology, Faculty of Medicine, Ardabil University of Medical Sciences, Ardabil, Iran; ^2^ Immunology Research Center, Tabriz University of Medical Sciences, Tabriz, Iran; ^3^ Stem Cell and Regenerative Medicine Institute, Tabriz University of Medical Sciences, Tabriz, Iran; ^4^ Student Research Committee, Tabriz University of Medical Sciences, Tabriz, Iran; ^5^ Department of Cancer and Inflammation Research, Institute of Molecular Medicine, University of Southern Denmark, Odense, Denmark; ^6^ Translational Research Institute (TRI), University of Queensland Diamantina Institute, The University of Queensland, Brisbane, QLD, Australia; ^7^ General and Vascular Surgery Department of Tabriz University of Medical Sciences, Tabriz, Iran; ^8^ Faculty of Medicine, Department of Immunology, Tabriz University of Medical Sciences, Tabriz, Iran; ^9^ Medical Oncology Unit, Istituto di Ricovero e Cura a Carattere Scientifico (IRCCS) Istituto Tumori “Giovanni Paolo II” of Bari, Bari, Italy; ^10^ Department of Biomedical Sciences and Human Oncology, Department of Internal Medicine and Oncology (DIMO), University of Bari, Bari, Italy

**Keywords:** myeloid-derived suppressor cells, tumor microenvironment, STAT3, TLR7/8, breast cancer

## Abstract

Cancer cells escape immune destruction. From this perspective, myeloid-derived suppressor cells (MDSCs), which are immunosuppressive in various cancers including breast cancer (BC), are significant. However, the precise mechanisms are unknown. We isolated HLA-DR^-^CD33^+^ MDSCs and CD3^+^ T cells from BC patients’ peripheral blood and healthy donors through MACS and immunophenotyped by flow cytometry. Transfection of short-interfering RNAs and treatment with a TLR7/8 agonist altered pathway activities *in vitro*. Gene expression was analyzed using qRT-PCR, western blotting, and immunohistochemistry. Our findings showed an association between the progression of BC and increased levels of circulating HLA-DR^-^CD33^+^ MDSCs. These cells strongly suppress both autologous and analogous CD3^+^ T cell proliferation and enter the tumor microenvironment. We also identified increased STAT3 signaling and increased IDO and IL-10 expression in BC-derived MDSCs as immunosuppression mechanisms. Further, STAT3 inhibition and TLR7/8 pathway stimulation reduce the immunosuppressive activity of patient-derived MDSCs on T cells by inducing MDSC repolarization and differentiation into mature myeloid cells. This also alters the expression of critical cytokines and transcription factors in CD3^+^ T cells and, importantly, reduces breast cancer cells’ proliferation. Finally, while chemotherapy is able to significantly reduce circulating MDSCs’ level in patients with breast cancer, these MDSCs remained highly T cell-suppressive. We identified a novel molecular mechanism of MDSC-mediated immunosuppression. STAT3 inhibition and TLR7/8 pathway stimulation in MDSCs repolarize and suppress MDSCs from breast cancer patients. This offers new opportunities for BC immunotherapy.

## Introduction

Following extensive immunological studies and significant pre-clinical and clinical findings over the years, researchers have found that the immune system has the crucial role of identifying and eliminating tumor cells (1, 2). Immunotherapy has been shown as a promising therapeutic approach, and numerous immunotherapeutic protocols are being tested or implemented in the clinic to specifically treat breast cancer (BC). Targeted immunotherapies, such as monoclonal antibodies, that aim at inducing an antitumor immune response are becoming primary treatment functions for BC. Accumulating evidence represents a promising result in cancer vaccinology. This immunotherapeutic protocol activates both the adaptive immune response and immunological memory. Encouraging results of BC vaccines are coming out during numerous clinical trials such as NeuVax, AVX901, and INO-1400. However, the efficacy of these therapeutic approaches has been severely impaired by tumor-associated immunosuppressive mechanisms (3–8). Several studies on mice and humans have shown the importance of the tumor immune microenvironment in the development and progression of cancer (9, 10).

Myeloid-derived suppressor cells (MDSCs) are known as a group of heterogeneous immune cells that mainly include immature myeloid cells (IMCs) and myeloid progenitors (11). Several studies on human and mouse cancer models have shown that the infiltration and induction of MDSCs in the tumor’s microenvironment are controlled *via* various factors such as inflammatory mediators, cytokines, and growth factors (12).

It is noteworthy that the increase in the production and activation of IMCs in cancer does not necessarily lead to the expansion of MDSCs. Two different mechanisms of expansion and activation are responsible for increasing MDSCs under pathological conditions. In cancer patients, the tumor cells release various mediators such as prostaglandins, vascular endothelial growth factor (VEGF), Bombina variegate peptide 8 (Bv8), etc. that impede the differentiation of mature myeloid cells and stimulate MDSCs expansion. Moreover, the important MDSC activating mediators such as Transforming Growth Factor (TGF-ß), IL-1β, IL-4, etc. induce their activation. They promote more flexibility in regulating these cells under pathological and physiological conditions (13). The importance of MDSCs in the development and progression of BC has been elucidated through studies on animal models. However, these models do not fully reflect the level of expression and the role of functional genes and proteins in the breast tissue of the human species (14, 15). Unlike murine MDSCs, which are highly distinct due to the expression of GR1 and CD11b molecules, human MDSCs have not been well-defined, owing to the lack of specific markers, and both the function of these cells and their relationship with clinical features of patients remain poorly understood (15).

In our previous study, we determined the frequency and phenotypes of MDSCs in peripheral blood samples of BC patients. We demonstrated that the presence and frequency of these circulating cells are associated with disease severity and prognosis and other clinicopathological characteristics of BC (16). In the current study, we investigated the prevalence and phenotype of MDSCs, as well as the blood samples and tissue samples of patients with BC before and after chemotherapy. Also, the immunosuppressive activity and differentiation of MDSCs isolated from breast cancer patients were examined before and after targeting the STAT3 transcription factor using siRNA in MDSCs along with simultaneous activation of the TLR7/TLR8 signaling using a specific agonist.

## Materials and Methods

### Patients and Ethics Statement

Blood samples were taken from patients (n=20) before start and after completion of chemotherapy and from age- and sex-matched healthy donors (n=12). The samples of pathologically diagnosed BC tissues paired adjacent tissues, and normal breast tissue was obtained from voluntary and healthy individuals. The aimed experiments were ultimately proved by clarification and written proof of consent from each case. All the cases considered were women with an average age of 47.2 years (from 29 to 73 years) who were histologically diagnosed with BC (**Table 1**). The present investigation was conducted based on the *amended declaration of Helsinki* principles, and it was confirmed by the Ethics Committee of Tabriz University of Medical Sciences (TBZMED.REC.1394.1129). It should be taken into account that none of the cases had a background of autoimmune disease or consumption of immunosuppressive drugs over the last three months. Also, none of them had a history of any other kind of cancer.

**Table 1:** Clinicopathological characteristics of breast cancer patients.

Characteristic	Category	Cases
Health status	Healthy Breast cancer	12 20
Age in the breast cancer group	≤45 year >45 year	11 9
Tumor size (cm)	<3 cm ≥3 cm	12 8
Clinical stage	Stage I Stage II Stage III Stage IV	5 5 5 5
Lymph node metastasis	Positive Negative	14 6
Tumor site	Right Left	9 11
Receptor status		
ER/PR (+) and Her-2/neu (−) ER/PR (−) and Her-2/neu (+) Triple-negative	Group1 Group2 Group3	11 5 4

### PBMC Isolation

Peripheral blood mononuclear cells (PBMCs) were isolated by the density gradient centrifugation method, as we previously described (17).

### Immunophenotyping of MDSCs

To characterize the MDSC populations in freshly isolated PBMCs, a range of antibodies were used, including HLA-DR, CD33, CD14, CD15, CD3, CD19, and CD56 (BioLegend, San Diego, CA, USA). Moreover, antibodies against the following surface antigens were used to evaluate MDSC differentiation: CD86, CD11c, and CD206. All stained samples and their respective isotype controls were incubated in PBS containing 0.5% bovine serum albumin (BSA) and FC-receptor blockers (BD Biosciences, San Jose, CA) to restrict non-specific binding sites. The results obtained were evaluated by a MACSQuant 10 Analyser (MiltenyiBiotec, Germany). A minimum of 10,000 live events per sample were acquired for analysis. Briefly, following the initial forward versus side scatter (FSC vs. SSC) discrimination, the gate was set on HLA-DR^−^/CD33^+^ cells. Next, we gated on the subpopulations defined as MDSC, including G-MDSC (HLA-DR^-^CD33^+^CD15^+^), M-MDSC (HLA-DR^-^CD33^+^CD14^+^) cells, and their combinations. Moreover, all data analyses were carried out using FlowJo software (Tree Star, Ashland, OR).

### MDSC and T Cell Isolation

Freshly provided PBMCs were applied for the enrichment of HLA-DR^–^ CD33^+^ MDSCs using MACS magnetic bead isolation kits (MiltenyiBiotec, Bergisch Gladbach, Germany) according to the manufacturer’s instructions. In brief, isolated PBMCs were incubated with anti-HLA-DR Ab-coated magnetic beads (Miltenyi) for 15 min in the refrigerator (2−8°C). Following incubation time, the cells were washed by adding 2 mL of buffer per 10^7^ cells and centrifuged at 300×g for 10 min, and resultant pellets were resuspended in buffer. Then, the cell suspension was applied onto an appropriate MACS Column and MACS Separator (Miltenyi). Negatively selected cells (HLA-DR^−^) were then incubated with anti-CD33 Ab-coated magnetic beads for 10 min to identify HLA-DR^−^/CD33^+^ cells positively.

The CD3^+^ T cell population (autologous and analogous) was further enriched from freshly isolated PBMCs using the human Pan T Cell Isolation Kit II (MiltenyiBiotec, Bergisch Gladbach, Germany) by negative selection based on the manufacturer’s protocols. After sorting, the cells were washed and stained to HLA-DR, CD33, and CD3 by adding anti-human monoclonal fluorescently labeled antibodies, and purity of sorted populations was recognized by flow cytometry. Cell purities more than 90% were implemented in the experiments.

### *In Vitro* Gene Silencing

Isolated MDSCs were transfected with siRNA using transfection reagent JetPRIME (Polyplus, Illkirch, France), at the final concentration (60 pmol) according to the manufacturer’s recommendations. For siRNA transfection of MDSCs, cells were plated onto a 24-well plate and incubated at 37°C overnight. Then, siRNAs and the reagents of siRNA transfection were subsequently diluted in siRNA transfection medium (sc-29493, Santa Cruz Biotechnology, CA, USA) individually. Afterward, they were incubated at room temperature (25°C) for 5 min. The supplied solutions were then mixed and incubated at room temperature (25°C) for 30 min. Before transfection, the medium was changed to Opti-MEM Medium, and then the mixtures were added to the cells in a drop-wise manner. Cells were incubated at 37°C for 5–6 h in a humidified atmosphere of 5% CO2, and then RPMI-1640 medium containing 20% FBS was added. The used sample of STAT3 siRNAs in this section of work included three different siRNA duplexes. Scrambled siRNA (Santa Cruz Biotechnology, Inc., Santa Cruz, CA) was considered as a negative control for the experiments (**Supplementary Table S1**).

### MDSC Co-Culture Experiment and Treatment

MDSCs were further treated with five µg/ml R848 (Resiquimod), which act as a TLR7/8 agonist, and were incubated at 37°C for three days in a humidified atmosphere of 5% CO_2_. Differentiation of the transfected and treated MDSCs was evaluated by flow cytometry using the mentioned antibodies. Moreover, the transfected and treated MDSCs were subdivided into different groups and co-cultured with T cells at different ratios in transwell inserts (Greiner Bio-one, The Netherlands) in RPMI-1640 medium, containing 10% fetal bovine serum (FBS; GIBCO, Carlsbad, CA, USA) and 1% penicillin/streptomycin (GIBCO, Carlsbad, CA, USA).

### MDSC Suppression Analysis

To evaluate CD3+ T cells’ proliferation, BrdU (Bromodeoxyuridine) was used, following the manufacturer’s instructions. T cells were co-cultured with MDSCs at the following ratios: 0:1, 1:1, and 1:2 in RPMI-1640 medium in transwell inserts. Anti-CD3/anti-CD28 antibodies (at bead/cell ratio of 1:1; Human T Cell Activator CD3/CD28 Dynabeads Invitrogen, Carlsbad, CA) was also supplied with 500 IU/ml of IL-2 (R&D Systems, Minneapolis, MN) and then applied to stimulate the CD3^+^ T cells. The levels of BrdU incorporation were calculated in T cells with a spectrophotometer at 450 nm.

### Immunohistochemistry (IHC)

BC tissues, paired adjacent tissues, and normal breast tissues were immediately fixed in a buffered formalin solution (10%) and embedded in paraffin. Subsequently, it was cut into 4-μm sections. The sections were deparaffinized and rehydrated in xylene and measured amounts of ethanol in water, respectively. Tissue slides were then subjected to antigen retrieval by sodium citrate buffer (10 mM sodium citrate, 0.05% Tween 20, pH 6.0). Hydrogen peroxide at 3% was used to quench endogenous peroxidase activity for 20 min. All specimens were immunostained using specific antibodies for CD33 (Abcam, Cambridge, MA, USA), pSTAT3 (Santa Cruz, CA, USA), STAT3 (Santa Cruz, CA, USA), and Ki67 (Bio Legend, San Diego, USA). Subsequently, a horseradish peroxidase-based detection system was used to identify positive cells (EnVision, DAKO). Finally, the images were assessed using an Olympus BX51 microscope (Olympus, Tokyo, Japan).

### RNA Extraction and qRT–PCR

Total RNAs in the treated and untreated cells (MDSCs and T cells) were isolated according to the manufacturer’s recommendations for RiboEx (GeneAll, GeneAll Biotechnology, Seoul, Korea). RNA was reverse-transcribed using the BIOFACT kit (Co, LTD, Korea), directed by the manufacturer. The qRT-PCR was used to determine the relative quantities of mRNA of the respective genes, including STAT3, IDO, ARG1, IL-12, IL-4, INF- γ, TLR7, TLR8, IL-17, IL-10, RORϒT, T-bet, GATA3, and FOXp3 using a SYBR Green I PCR Master Mix (BIOFACTco., Daejeon, Korea). All primer sequences were listed in **Supplementary Table S2**. Samples were run on the Roche lightCycler^®^ 96 Real‐Time PCR system (Roche Diagnostics, Mannheim, Germany). Data were independently normalized to the housekeeping gene S18 mRNA expression for each sample, and fold change was calculated using the 2^(-ΔΔCt)^ method (18).

### Western Blotting

MDSCs were detached and lysed with RIPA buffer (Santa Cruz Biotechnology, Santa Cruz, CA) containing protease inhibitors. Proteins were separated on polyacrylamide-SDS gels using Mini Protean TGX 4–15% gels (Bio-Rad), electro-blotted from gel to a polyvinylidene difluoride (PVDF) membrane (BioRad). The membranes were also blocked with phosphate-buffered saline-Tween 20 (PBS-T) and incubated with specific primary antibodies against STAT3 (cat. no. sc-482) and pSTAT3 (cat. no. sc-7993) at 4°C overnight. Then, the protein bands were probed with HRP-conjugated secondary antibodies (Santa Cruz). A mouse anti‐β‐actin antibody was used as an internal control. ECL prime western blot reagent (Amersham) was also considered in this experiment as chemiluminescence substrates. ImageJ software was implemented for the evaluation and qualification of immunoblots.

### Statistical Analysis

Experiments were performed with 20 patients and 12 healthy samples as indicated and were performed in triplicate. Graph Pad Prism (Graph Pad Software, La Jolla, CA, USA) was used for statistical analyses. All data were expressed as the mean ± standard deviation (SD). Parametric or non-parametric tests as required by the variable distribution, the Student’s t-test and one-way ANOVA for normally distributed variables, and the Mann–Whitney test for variables that were non-normally distributed were used to assess the differences between the groups examined. Furthermore, they were defined for multiple testing by applying q-values based on the previous report (Benjamini and Hochberg method), calculating a false discovery rate (FDR) of 5%. *P*- and *q*-values <0.05 were considered statistically significant.

## Results

### BC Progression Is Associated With an Accumulation of Circulating HLA-DR^–^ CD33^+^ Cells

**Figure 1:**
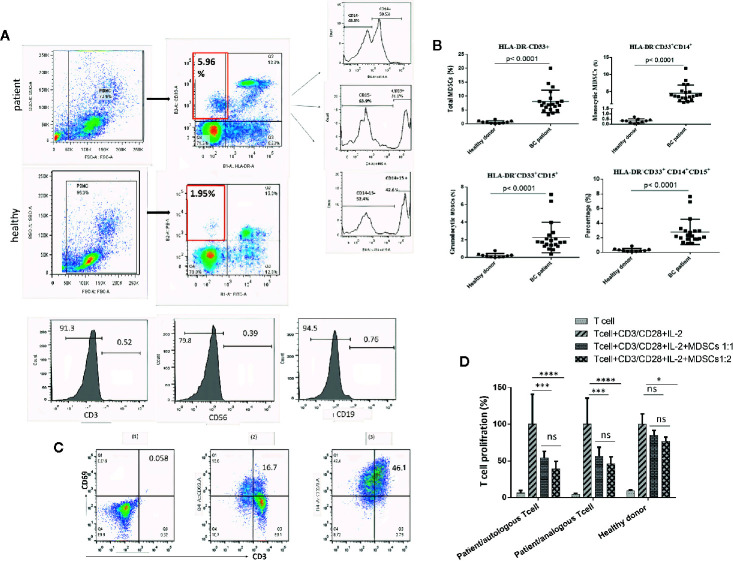
Circulating HLA‐DR^‐^CD33^+^ myeloid‐derived suppressor cells in BC patients. The frequencies and phenotypes of the studied MDSCs in patients’ blood samples and healthy cases were assessed using multi-color flow cytometry. **(A)** Shows the characterization and quantification of MDSCs as the HLA‐DR^-/low^ CD33^+^ population and as a percentage of gated cells *via* supplying a minimum of 10,000 live events per sample. Histogram plots also indicate the expression rate and percentages of the positive cells for surface markers (CD14 and CD15) on gated HLA‐DR^-/low^ CD33^+^ cells. HLA‐DR^-/low^ CD33^+^ cells were negative for the expression of lineage markers CD3, CD19, and CD56. **(B)** Percentages of MDSC subpopulations, including total MDSCs, monocytic MDSCs, and granulocytic MDSCs in peripheral blood of the study groups (*P* < 0.0001). The calculated bars reveal the mean ± standard deviation (SD) of each studied group. **(C)** Anti-CD3/anti-CD28 antibodies and IL-2 stimulated t cells to proliferate. The expression of CD69 as a T cell activation marker was quantitatively measured by flow cytometry; (1) unstained, (2) T cells without CD3/CD28 and IL-2, (3) T cells stimulated with CD3/CD28 and IL-2. **(D)** Reveals the percentage of T cell proliferation when co‐cultured with various ratios of MDSCs; means ± standard deviation, *p = 0.0024, ***p = 0.0002, ****p < 0.0001. BC, breast cancer; HD, healthy donor; PBMC, peripheral blood mononuclear cell; MDSC, myeloid‐derived suppressor cells; IL‐2, interleukin‐2; ns, not significant.

Our previous study established the immunophenotyping of MDSCs as HLA-DR^-^CD33^+^ cells in patients with BC (17). Flow cytometry scatter plot results, based on the gating strategy, are indicated for one of the patients and one of the healthy donor samples that participated in our study. Further phenotypic characterization indicated that, compared to healthy individuals, in BC patients, not only the total fraction of circulating MDSCs (HLA-DR^-^ CD33^+^ cells) is increased, but the fractions of each of the MDSC subpopulations, including G-MDSCs (HLA-DR^-^ CD33^+^ CD15^+^) and in particular M-MDSCs (HLA-DR^-^ CD33^+^ CD14^+^), are also elevated. Moreover, we observed that HLA-DR^-^ CD33^+^ cells are negative for the lineage markers expressions (CD3, CD19, and CD56) (**Figure 1A**). The percentages of total MDSCs, M-MDSCs, and G-MDSCs for BC patients were 8.08 ± 4.04%, 4.56 ± 2.41%, and 2.27 ± 1.72%, while for healthy donors they were 0.68 ± 0.48%, 0.23 ± 0.22%, and 0.31 ± 0.19%, respectively; all these percentages are considered as statistically significant differences (each *P*<0.0001) (**Figure 1B**). These results suggested that circulating M-MDSCs may have an essential role in the development and progression of BC.

T cells treated with anti-CD3/anti-CD28 antibodies and IL-2 have shown high expression of CD69 as a T cell activation marker, which was quantitatively measured by flow cytometry (**Figure 1C**). To confirm that the HLA-DR^-^ CD33^+^ cells are MDSCs, tests were carried out on the suppressive effects of isolated cells on activated T cell proliferation. As shown in **Figure 1D**, compared to healthy donors, the purified HLA-DR^-^ CD33^+^ cells from the cancer patients significantly reduced autologous and analogous CD3^+^ T cell proliferation.

### MDSCs in Blood Circulation and Tissue of BC Patients Show Increased STAT3 Activity

**Figure 2:**
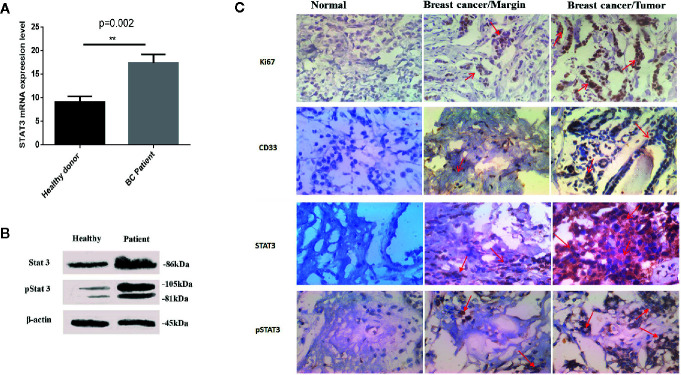
Both total and activated STAT3 levels are increased in MDSCs from BC patients. **(A)** Expression of STAT3 in MDSCs from healthy individuals and patients with BC. The results were presented as the mean expression level of STAT3 in each group. **(B)** STAT3 and pSTAT3 protein expression in MDSCs from healthy subjects and patients with BC. Patients with BC exhibit higher expression of the active form of STAT3. Beta-actin was used as a loading control. **(C)** Histological specimens from patients and healthy subjects were stained and evaluated by IHC for CD33, STAT3, pSTAT3, and Ki67 markers with relevant antibodies; arrows represent cells positive for studied markers. The results showed an increased level of the filtration of CD33^+^ STAT3^+^ p-STAT3^+^ cells into tissue samples of patients with BC; **P = 0.002.

To better illustrate the molecular mechanisms regulating MDSCs differentiation and immunosuppressive activities, we evaluated the total and the phosphorylated STAT3 expressions, both at mRNA and at protein levels, in circulating MDSCs using quantitative real-time PCR and western blotting analyses. HLA-DR^-^ CD33^+^ cells isolated from healthy donors were considered as normal myeloid-derived cell controls. As shown in **Figures 2A, B**, increased STAT3 expression, both at the mRNA and protein levels, was detected in patient MDSCs as compared to that in HLA-DR^-^ CD33^+^ controls. Notably, compared to HLA-DR^-^ CD33^+^ controls, a significant increase in phosphorylated STAT3 protein was detected in circulating MDSCs (**Figure 2B**). Thus, compared to healthy donor MDSCs, not only were the total STAT3 protein levels increased in BC patient-derived MDSCs, but STAT3 phosphorylation and activation were also considerably higher in BC patients. This reflects the importance of pSTAT3 signaling in differentiation and immunosuppressive activities.

We further investigated whether STAT3 and pSTAT3^+^ myeloid cells exist in circulation and in the BC microenvironment of breast tumors. To this aim, we evaluated the frequency of STAT3^+^, pSTAT3^+^, and CD33^+^ myeloid cells in BC tissues and IHC controls. Normal tissue adjacent to the tumor and normal breast tissues were regarded as a control. Microscopic analysis showed a higher level of cells that were positive for STAT, pSTAT3^+^, and CD33^+^ in BC tissues compared to tumor-adjacent normal tissues and normal breast tissue (**Figure 2C**), suggesting the passage of MDSCs from blood circulation into the BC tumor microenvironment.

### BC-Associated MDSCs Express Increased Levels of IDO and IL-10

**Figure 3:**
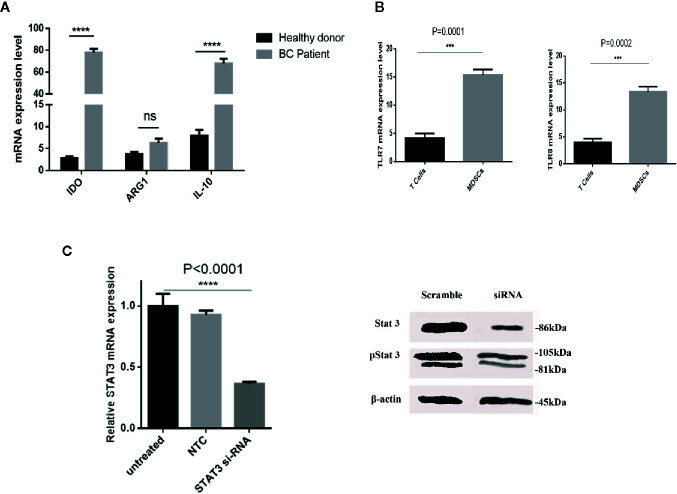
The immunosuppressive mechanism in BC-associated-MDSCs. **(A)** BC-associated-MDSCs have significantly higher expression levels of IDO and IL-10 genes than those of healthy subjects. No significant changes were observed in ARG1 gene expression, ****P < 0.0001. **(B)** The normalized expressions of TLR7 and TLR8 in the isolated MDSCs from the BC blood samples were investigated. The expressions of the considered genes in T CD3^+^ cells were used as negative controls. **(C)** Silencing of STAT3 by siRNA in BC-associated-MDSCs reduces the STAT3 and pSTAT3 protein expression. STAT3 gene expression is shown in treatment and control groups. Beta-actin was used as a loading control. STAT3 gene expression is shown in treatment and control groups. ***P < 0.001; ns, not significant.

We sought to establish the molecular basis of suppression mediated by patient-derived MDSCs. To this aim, relative mRNA expression of ARG1, IDO, and IL-10 genes in HLA-DR^-^ CD33^+^ cells purified from BC patients and healthy donors were compared. Based on the qRT-PCR results, there were significantly higher IDO and IL-10 mRNA levels in HLA-DR^-^ CD33^+^ cells sorted from BC patients compared to control subjects (**Figure 3A**). No significant difference in mRNA expression of ARG1 in HLA-DR^-^ CD33^+^ cells from patients relative to control subjects was observed. Our results showed that IDO and IL-10 mRNA levels were increased about 27.99 fold and 8.54 fold in cancer patients’ cells over control cells, respectively, but ARG1 levels were increased by 1.68 fold (**Figure 3A**). Our findings established MDSC IDO and IL-10 expression as a key regulator of the immunosuppressive characteristic in the BC-derived MDSCs. Thus, by driving IDO and IL-10 overexpression, breast tumors have the potential to induce an immunosuppressive microenvironment that inhibits the antitumor immune response.

### TLR7/8 Agonist and si-STAT3 Treatment Re-polarized BC-Associated MDSCs

**Figure 4:**
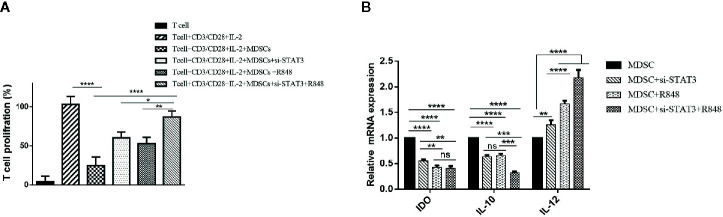
Reducing the immunosuppressive activity of MDSCs on T cells by inhibiting STAT3 expression and treatment with TLR7/8 agonist. **(A)** The proliferation of T cells co-cultured with MDSCs treated with si-STAT3 alone and/or TLR7/8 agonist was evaluated using the BrdU incorporation method. **(B)** Targeting of STAT3 with siRNA, as well as stimulation of cells with the TLR7/8 agonist R848, inhibits the expression of IDO and IL-10 genes and induces IL-12 in MDSCs isolated from patients with breast cancer. *P < 0.05 **P < 0.006, ***P < 0.0005, ****P < 0.0001; ns, not significant.

Several studies have described STAT3 as an essential factor for the suppressive activity of MDSCs, and inhibition of STATs blocks this effect. Moreover, it was documented that murine MDSC expresses TLRs and responds to stimulation by the TLR agonists. These findings led us to assess whether the behavior of BC-associated MDSCs might also be affected by TLR agonists. At first, we evaluated the expression level of TLR7 and TLR8 in isolated patient MDSCs (**Figure 3B**). Moreover, the expression level of STAT3 mRNA and protein after siRNA treatment was evaluated in BC-associated-MDSCs. Silencing of STAT3 by siRNA in BC-associated-MDSCs reduces the STAT3 and pSTAT3 protein expression (**Figure 3C**). Then, we decided to adopt a co-culture set-up in which CD3^+^ T cells treated with CD3/CD28 monoclonal antibody (mAb) were co-cultured with STAT3 siRNA or TLR7/8 agonist treated MDSCs alone or with combined STAT3 siRNA and TLR7/8 agonist. After three days of incubation, the suppressive function of treated and untreated MDSCs was evaluated using BrdU proliferation assay. As shown in **Figure 4A**, we observed that the proliferation of CD3^+^ T cells in the treated group compared to control was not only suppressed, but the proliferation of T cells was also increased in co-culture with treated MDSCs. These results indicate that treatment with si-STAT3 or TLR7/8 agonist alone or si-STAT3 plus TLR7/8 agonist abolishes the suppressive function of MDSCs.

This effect may be due to the acquisition of an antigen-presenting phenotype with the competence to induce T cell proliferation. Therefore, to investigate the stimulatory potential of treated MDSCs, we also assessed these cells’ phenotype using flow cytometry. At first, we measured IDO, IL-10, and IL-12 mRNA levels in the treated and untreated group MDSCs. Interestingly, the results proved that the expression level of IL-10 and IDO was increased in untreated MDSCs (*P*<0.0001). In contrast, in treated MDSCs, IL-12 gene expression was significantly higher than the untreated group (*P*<0.0001) (**Figure 4B**).

**Figure 5:**
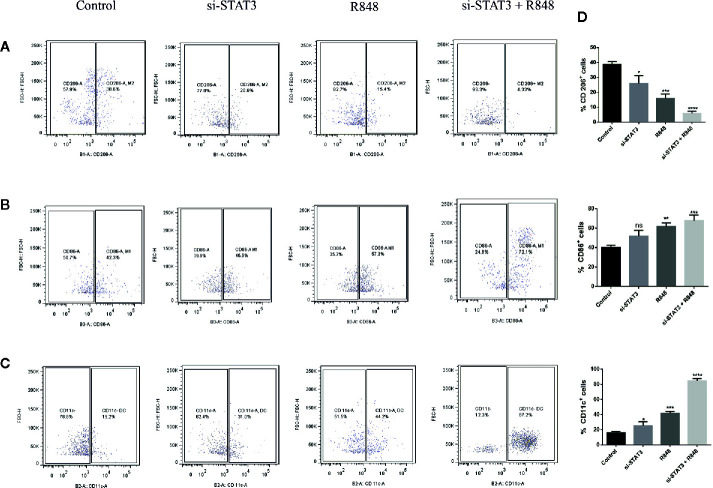
TLR7/8 agonist induces differentiation of MDSCs into mature myeloid cells. **(A–C)** The expression of CD206, CD86, and CD11C markers on MDSCs isolated from patients with BC was evaluated by flow cytometry. The ratio of the expression levels of these markers (right) and flow cytometric analyses (left) after MDSCs treatment with si-STAT3 and/or R848 are shown. The percentages of MDSCs expressing the markers CD206 **(A)** CD86 **(B)** or CD11c **(C)** after treatment with RPMI-1640 (control), si-STAT3, and R848 are shown, respectively. **(D)** Quantification of the expression of these markers. Results are shown as mean ± standard deviation. *P < 0.05, **P < 0.006, ***P < 0.0005, ****P < 0.0001; ns, not significant.

A previous study revealed that MDSCs express some M1 markers and some M2 markers, indicating that these cells are pleiotropic in terms of the M1 and M2 categories. It was also suggested that MDSCs resemble deactivated monocyte/macrophages, as they express the CD206 marker. Moreover, dendritic cells, as the significant antigen-presenting cells (APCs) among specialized APCs, are usually characterized as cells expressing CD11c. Hence, in the present study, polarization was assessed in the treated and untreated MDSCs by expression of surface antigens, including CD86, CD206, and CD11c, which are M1, M2 macrophages, and DC markers, respectively. Flow cytometric analysis revealed that BC-associated-MDSCs treated with si-STAT3 and TLR7/8 agonist expressed high levels of CD86 and CD11c, which is a sign of M1 polarization, but they expressed low levels of CD206 (**Figures 5A–D** and **Table 2**).

**Table 2:** Expression of surface markers of macrophages and dendritic cells in untreated and treated MDSCs.

Markers	Control	si-STAT3	R848	si-STAT3 +R848
**CD206**	38.6%	20.6%	15.4%	4.33%
**CD86**	42.3%	46.9%	57.3%	72.1%
**CD11c**	15.2%	31.0%	44.2%	87.2%

### TLR7/8 Agonist and si-STAT3 Treatment Repolarize T CD3^+^ Cells

**Figure 6:**
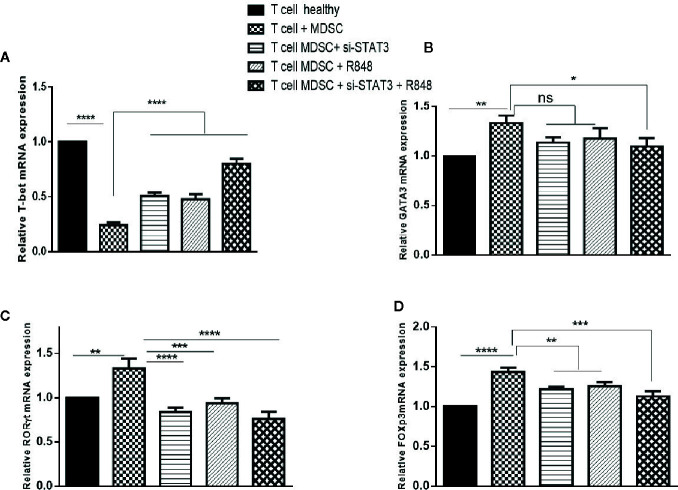
Co-culture with MDSCs changes the expression patterns of transcription factors in T CD3^+^ cells. The mRNA expression level of the **(A)** T-bet, **(B)** GATA3, **(C)** RORɤt, and **(D)** FOXP3 in the T CD3^+^ cells co-cultured with MDSCs treated with or without si-STAT3 and R848 were measured using qRT-PCR. The expression of these genes in T CD3+ cells isolated from a healthy subject was used as controls. Results are shown as mean ± standard deviation. *P < 0.03, **P < 0.008, ***P < 0.0004, ****P < 0.0001; ns, not significant.

Abolition of the inhibitory effect of TLR7/8 agonist and si-STAT3 treated MDSCs and their differentiation to adopt a pro-inflammatory phenotype prompted us to hypothesize that these differentiated MDSCs induce T CD3^+^ cell polarization. Hence, we first evaluated Th1, Th2, Th17, and Treg (T-helper cell subtypes) expression levels. Specifically, we assessed the expression levels of T cell-specific transcription factors, including T-bet, GATA3, RORγt, and the forkhead transcription factor FOXP3, in T cells in the studied groups. Treatment experiments showed that the expression of T-bet was increased (**Figure 6A**). However, compared to T cells co-cultured with untreated MDSCs, the expression of RORγt and FOXP3 was decreased in T cells co-cultured with treated MDSCs (*P*<0.0001) (**Figures 6C, D**). However, we observed no significant differences in the GATA-3 mRNA expression of the treatment group with si-STAT3 or TLR7/8 agonist alone, compared to the co-cultured T cells with untreated MDSCs, but the treated group with si-STAT3 plus TLR7/8 showed significant differences relative to the co-cultured T cells with untreated MDSCs (**Figure 6B**).

**Figure 7:**
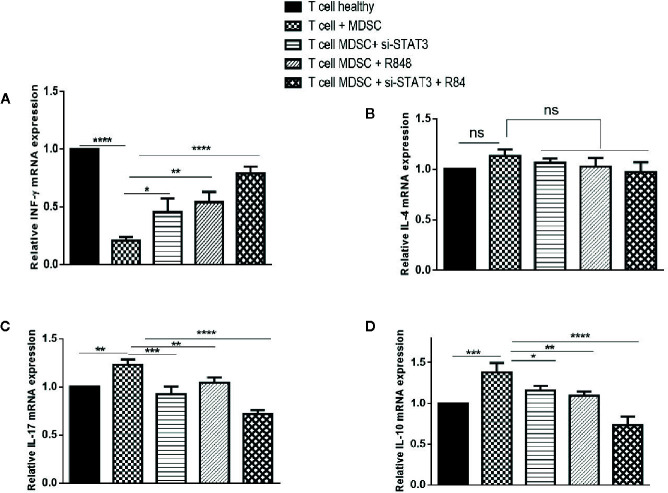
Co-culture with MDSCs changes expression patterns of cytokines in T CD3^+^ cells. The mRNA expression levels of INF-ɤ **(A)**, IL-4 **(B)**, IL-17 **(C)**, and IL-10 **(D)** in T CD3^+^ cells co-cultured with MDSCs treated with si-STAT3 and/or R848 were measured using qRT-PCR. The expression of these genes in T CD3+ cells isolated from a healthy subject was used as controls. Results are shown as mean ± standard deviation. *P < 0.03, **P < 0.008, ***P < 0.001, ****P < 0.0001; ns, not significant.

We also analyzed cytokines’ expressions, including IL-4, IL-17, INF-γ, and IL-10 in each condition. Treatment experiments showed that, compared to the co-cultured T cells with untreated MDSCs, the expression of INF-γ was elevated, whereas the expression of IL-17 and IL-10 was reduced in T cells co-cultured with treated MDSCs (*P*<0.0001). In contrast, IL-4 levels revealed no considerable difference in the same treated group compared to the control group (**Figures 7A–D**).

### TLR7/8 Agonist and si-RNA STAT3 Treatment Reduce the Proliferation of BC Cells

**Figure 8:**
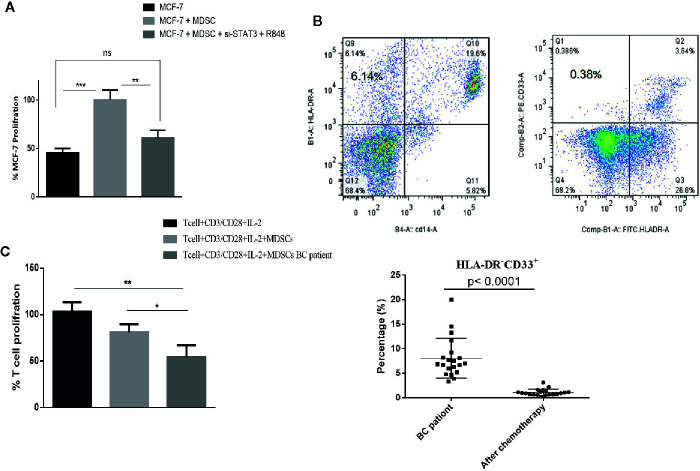
In patients with BC treated with chemotherapy, circulating MDSC levels are decreased, but their MDSCs remain highly T cell-suppressive. **(A)** In a co-culture experiment, treatment of MDSCs with the TLR7/8 agonist R848 and si-STAT3 reduces MCF-7 BC cells’ proliferation. **(B)** The frequency of MDSCs in BC patients undergoing chemotherapy was assessed. The flow cytometric results showed that after chemotherapy (right), the circulating MDSC frequencies were decreased. **(C)** The MDSCs of patients with BC after chemotherapy still had more T cell-suppressive activity than HLA-DR^-^CD33^+^ MDSCs isolated from healthy individuals. *P < 0.05, **P < 0.003, ***P < 0.0005; ns, not significant.

Many studies investigating the interactions between MDSCs and tumor cells have shown that the presence of MDSCs in the tumor microenvironment plays an important role in tumor growth. To evaluate the potential effect of differentiated MDSCs on tumor growth and proliferation, MCF-7 cells with MDSCs that were treated with or without si-STAT3 and the TLR7/8 agonist R848 were co-cultured and MCF-7 proliferation was assessed using the BrdU incorporation method. This revealed that si-STAT3 and R848 treatment of MDSCs significantly reduces the proliferation of MCF-7 tumor cells (**Figure 8A**).

### Chemotherapy Reduces the Level of Circulating MDSCs in BC Patients, but These MDSCs Remain Highly T Cell-Suppressive

Several recent studies have also reported different effects of chemotherapy on the MDSCs. Therefore, we assessed the frequency and function of MDSCs in patients with BC undergoing chemotherapy. Peripheral blood specimens were collected from patients before and after chemotherapy. Immediately following PBMCs separation, the frequencies of MDSCs were analyzed by flow cytometry. This showed that after chemotherapy, the frequencies of circulating MDSCs were decreased (1.07 ± 3.62%) (**Figure 8B**). However, it is of note that the MDSCs of patients with BC after chemotherapy still had considerably more T cell-suppressive activity than HLA-DR^-^CD33^+^MDSCs collected from healthy individuals. Specifically, the co-cultured T cells’ proliferation fractions with MDSCs were 80.89% and 53.85%, respectively (**Figure 8C**).

## Discussion

In our previous study, the frequency and significant phenotype of the circulating MDSCs taken in consideration were assessed in the patients with BC at various stages of the disease (17). The significance of our study on cancer treatment is that it can reduce tumor cell escape from the immune system and the immunosuppressive effects of MDSCs. MDSCs play an important role in affecting the effectiveness and efficiency of chemotherapy drugs, and these cells themselves are also affected by these chemotherapy drugs. Studies on animal and human models have revealed that chemotherapeutic agents may have several, sometimes conflicting, effects on MDSCs. These contradictions may be due to various factors, such as the type of animal model or the type of tumor (19). This suggests a necessity for more information regarding effective targeting of MDSCs and about functional markers in these cells and the tumor microenvironment.

Highfill et al. showed that the suppression of the antitumor immune response by MDSCs could be significantly inhibited by controlling the CXCR2 factor, which is involved in the migration of MDSCs to the tumor tissue (20). Another study identified that B7-H4 is a functional marker for MDSCs in humans and mice, and its inhibition could decrease the capacity of immunosuppression in these cells (21). Based on the findings from a range of studies, including this study, it can be concluded that intracellular signaling mediators involved in immunosuppressive activities can be considered as functional biomarkers for MDSCs.

The behavior of MDSCs is regulated by transcription factors (22). We and others have identified the STAT3 transcription factor involved in MDSCs recruitment, activation, and suppressive function (23, 24). Numerous STAT3-activating factors are available in the tumor microenvironment. Also, through a positive feedback loop, some genes induced by STAT3 promote continuous activation of the STAT3 pathway (25). Notably, activation of STAT3 occurs in cancerous cells and in immune cells, such as tumor-infiltrating MDSCs as shown here. STAT3 promotes inter-cellular interactions in the tumor microenvironment. It was proposed that STAT3 activation is significantly involved in differentiating myeloid cells into immunosuppressive myeloid cells, thus making STAT3 an important target (26). In the Smo*mouse model, removal of STAT3 in myeloid cells by the Cre/LoxP system led to increased macrophages with pro-inflammatory phenotype and a critical reduction in G-MDSCs. In addition, in these mice, the ratio of effector T-cells to regulatory T-cells was elevated. Here, our present study sheds new light on this, as we showed that pSTAT3 levels in circulating MDSCs are elevated compared to MDSCs from healthy individuals. Also, CD33^+^, STAT3^+^, and pSTAT3^+^ cells in BC tissue are significantly higher in the tumor tissues compared to the tumor margin and absent or low in healthy controls.

In addition to established STAT3 activation mechanisms, we also studied the expression of ARG1, IDO, and IL-10, which are related to the functional mechanism of the MDSCs. ARG1 expression results in the consumption of amino acids, L-arginine, and L-cysteine ​in the tumor microenvironment (27). Serafini et al. used a B-cell lymphoma model to link the expression of ARG1 to MDSCs proliferation and expansion (28). However, in our study, there was no significant increase in the level of ARG1in patient-derived MDSCs. A study on prostate cancer indicated that ARG1 and IDO alter intratumoral CD8^+^ T cells’ functions in plasmacytoid DCs simultaneously. However, the role of IDO has not previously been investigated in MDSCs. In this study, we show that IDO is increased in BC-derived MDSCs. Together, these results suggest that some immunosuppression programs in different myeloid cells are shared in cancers (29, 30). Simultaneously, the expression of IDO in myeloid-like cells in tumor stroma isolated from patients with BC (31) was confirmed. Thus, there are probably cancer type-specific mechanisms.

Aside from increased IDO expression, our study showed that IL-10 in MDSCs isolated from patients increased significantly compared to CD33^+^ cells isolated from healthy individuals. This suggests that the suppressive mechanism of MDSCs in BC is dependent on the increased expression of both IDO and IL-10, but not the ARG1 enzyme. In contrast, the immunosuppressive activity of G-MDSCs was shown to be dependent on the ARG1 expression (32). These results were in accordance with the observation that M-MDSCs levels in patients were significantly higher than G-MDSCs. This demonstrates the multifaceted complexity of STAT3-mediated target gene expression.

The higher expression of IDO by M-MDSC in CLL (chronic lymphocytic leukemia) was reported, and its inhibition leads to an increase in T cell proliferation (33). Similar results were reported in a post-allograft study, demonstrating that M-MDSCs employ their immunosuppressive effects through the production of IDO (34). On the other hand, the suppressive activity of M-MDSCs was shown to be independent of ARG1 and IDO in DLBCL (diffuse large B-cell lymphoma). Our results were consistent with the results of Yu et al., which showed that IDO and pSTAT3 expressions in the collected MDSCs from the patients with BC had increased, and this correlated with infiltration of Treg cells into tumor tissue (35). Several studies, like this one, have designated targeting STAT3 in MDSCs as an anticancer strategy. Meanwhile, several other studies have shown that various drugs that target STAT3 or eliminate MDSCs, such as 5FU and doxorubicin, cannot be considered as an effective therapeutic strategy alone (36). Since MDSCs are critical suppressors of antitumor immune responses, and elimination of these cells enhances cancer vaccine efficacy, cancer therapies that target these cells along with conventional protocols may be more effective. Alternatively, instead of combining the treatments, triggering T cell activation and simultaneously targeting MDSCs improve therapeutic efficacy. In this regard, it is noteworthy that MDSCs can be differentiated into stimulatory APCs through the effect of cytokines likeIL-12, TLR9 ligands, or CPG conjugated to si-STAT3.

Zoglmeier et al. determined that TLR9 and TLR3 agonists, CPG, and Poly I:C respectively, can decrease the MDSCs suppressive functions and enhance MDSC differentiation (37). Paradoxically, a TLR4 agonist, lipopolysaccharide (LPS), increases the suppressive function of MDSCs (38). Accordingly, it has been postulated that this dual role is due to cross-reactivity with other active signaling pathways in normal cells or inflammatory conditions induced by the tumor (39, 40). TLRs signaling may be accompanied by negative regulatory feedback from the STAT3 pathway. For instance, Hossein et al. used a mouse model showing that pre-existing STAT3 in myeloid cells that contribute to tumors such as MDSCs can considerably direct the TLR9 signaling process to the tumor angiogenesis and inhibition of antitumor immune responses (41).

At this point, our implication of TLR7/8 signaling in the immunosuppressive function of MDSCs associated with BC is a novelty. In this study, we studied TLR7/8 signaling for the first time by stimulating it using a specific agonist, Resiquimod, in MDSCs isolated from BC patients. Also, we studied the role of this agonist along with STAT3 pathway inhibition. Spinetti et al. indicated that intra-tumor administration of R848 to mice with colon tumors significantly reduced the number of MDSCs (42). Our flow cytometry results showed that the activation of TLR7/8 and inhibition of STAT3 significantly induced differentiation of MDSCs in cells with an APC phenotype, such as macrophages and dendritic cells. In addition, this led to a loss of their suppressive function. Co-culture of tumor cells with reeducated MDSCs showed inhibition of tumor cell proliferation in treated groups, suggesting that the M1 phenotype induced in MDSCs might account for the inhibition of tumor cell proliferation. Our results are consistent with the result of a published article reporting that human CD34^+^ bone marrow cells are differentiated by R848 into different myeloid cells such as activated macrophages and myeloid dendritic precursors (43). The characterization of these results by following markers CD11c, CD13, and CD14 indicated that TLRs signaling might regulate bone marrow cells’ development.

Evaluation of the pattern of gene expression in T cells co-cultured with MDSCs also yielded new insights. The differentiated MDSCs caused a shift of T cells to TH1 and TH17. In line with this, Wogher et al. documented that stimulation of TLR7/8 signaling induced the production of TH1 cytokines (43). On the other hand, it was indicated that at the tumor site, the increase in MDSCs is commonly accompanied by the accumulation of Th17 cells. Th17 cells may also be detected in tumor lesions. In animal models, IL-17 was required for the development and tumor-promoting characteristics of MDSCs (44, 45).

In the present study, we also showed that MDSCs were decreased in patients after chemotherapy. However, there was no significant difference in these cells’ suppressive function compared to samples examined before chemotherapy. Therefore, the efficacy of immunotherapy and chemotherapy may significantly improve by reprogramming MDSCs into cells with an APC phenotype.

Our work indicates that disruption of the vicious circle involving STAT3 signaling in the tumor microenvironment, along with the stimulation of TLR7/8 signaling, could induce effective antitumor immune responses without toxicity and chemotherapy-associated side effects. Thus, combined stimulation of TLR7/8 signaling with the Resiquimod agonist, along with inhibition of STAT3 signaling, can be used as a potential therapeutic strategy for BC immunotherapy, either individually or combined with other immunotherapies, such as inhibition of immune checkpoints and adoptive T cell therapy.

## Data Availability Statement

The raw data supporting the conclusions of this article will be made available by the authors, without undue reservation.

## Ethics Statement

The studies involving human participants were reviewed and approved by Ethics Committee of Tabriz University of Medical Sciences (TBZMED.REC.1394.1129). The patients/participants provided their written informed consent to participate in this study.

## Author Contributions

ES designed and performed experiments and wrote the paper. AM performed molecular experiments. BM performed flow cytometry experiments. PD and AD helped write the manuscript and analysis data. SH contributed to clinical sample and data preparation. VK. performed data analyses. TK performed cellular experiments. NS and BB supervised the research, designed experiments, and helped write the manuscript. All authors contributed to the article and approved the submitted version.

## Funding

This project was funded by the Immunology Research Center of Tabriz University of Medical Sciences (Grant#94/09), and the authors are grateful for its support.

## Conflict of Interest

The authors declare that the research was conducted in the absence of any commercial or financial relationships that could be construed as a potential conflict of interest.
