# Supplementary material for: STAT3 Silencing and TLR7/8 Pathway Activation Repolarize and Suppress Myeloid-Derived Suppressor Cells From Breast Cancer Patients
Source: Front Immunol. 2021 Feb 19;11:613215. doi: 10.3389/fimmu.2020.613215 (PMC7933669; doi:10.3389/fimmu.2020.613215)
Supplement: Supplementary file 1 [file Table_1.docx]

**Supplementary Table1. Three different STAT3 siRNA sequences.**

| **siRNA duplexes** | **Sense** | **Antisense** | |
| --- | --- | --- | --- |
| **STAT3 siRNA (h) A** | **GAGACAUGCAAGAUCUGAATT** | | **UUCAGAUCUUGCAUGUCUCTT** |
| **STAT3 siRNA (h) B** | **GGAUCCCGGAAAUUUAACATT** | | **UGUUAAAUUUCCGGGAUCCTT** |
| **STAT3 siRNA (h) C** | **CCUCUCUGCAGAAUUCAAATT** | | **UUUGAAUUCUGCAGAGAGGTT** |
