# Supplementary material for: STAT3 Silencing and TLR7/8 Pathway Activation Repolarize and Suppress Myeloid-Derived Suppressor Cells From Breast Cancer Patients
Source: Front Immunol. 2021 Feb 19;11:613215. doi: 10.3389/fimmu.2020.613215 (PMC7933669; doi:10.3389/fimmu.2020.613215)
Supplement: Supplementary file 2 [file Table_2.docx]

**Supplementary Table 2. Primer and target sequences**

|  | **Sequence 5ʹ ̶ > 3** | **Gene** |
| --- | --- | --- |
|  | F: CACCTTTGACATGGAGTTGACC  R: AGCAGATCACCCACATTCACT | STAT3 |
|  | F: AGCTTATGACGCCTGTGTGAA  R: TCCTTTGGCTGCTGGCTTG | IDO |
|  | F: ACCTTGGCTTGTTTCGGACTT  R: ACACCAAGAGGGAATTTGTAGAGT | ARG1 |
|  | F: TCAGAATTCGGGCAGTGACTATTG  R: ATCCTTCTTTCCCCCTCCCTA | IL-12 |
|  | F: ACAGGCACAAGCAGCTGATC  R: AGCCTTTCCAAGAAGTTTTCCAA | IL-4 |
|  | F: TGTCGCCAGCAGCTAAAACAG  R: CCTAGTTGGCCCCTGAGATAAAG | INF- γ |
|  | F: AATGTCACAGCCGTCCCTAC  R: TTATTTTTACACGGCGCACA | TLR7 |
|  | F: TCCTGTGAGTTATGCGCCG  R: GTAGTCGACGATTGCTGCACT | TLR8 |
|  | F: TCTGTGATCTGGGAGGCAAAG  R: CTGGGTCGGCTCTCCATAG | IL-17 |
|  | F: AGGAAGAGAAACCAGGGAGC  R: GAATCCCTCCGAGACACTGG | IL-10 |
|  | F: TCGGAGCCTGTGTAGCCA  R: CAGGTGACTCGGTTTCAGTGC | RORϒT |
|  | F: TCTCCTCTCCTACCCAACCAG  R: CATGCTGACTGCTCGAAACTCA | T-bet |
|  | F : GCATCCAGACCAGAAACCGAA  R: TCGCGTTTAGGCTTCATGATACT | GATA3 |
|  | F: CACAACCTGAGTCTGCACAAGT  R: GTTCGTCCATCCTCCTTTCCTT | FOXp3 |
|  | F: GATCAGATACCGTCGTAGTTCC  R: CTGTCAATCCTGTCCGTGTC | 18S |
